# Supplementary material for: A Likelihood Approach for Real-Time Calibration of Stochastic Compartmental Epidemic Models
Source: PLoS Comput Biol. 2017 Jan 17;13(1):e1005257. doi: 10.1371/journal.pcbi.1005257 (PMC5240920; doi:10.1371/journal.pcbi.1005257)
Supplement: S1 File — (TAR.GZ) [file pcbi.1005257.s014.tar.gz › HSPH_Online-SI-Revision/PF08/n1-mild/n1-mild_PF_table0.pdf]

1	R0	{1.27677, 7.89375}	{1.72152, 14.9874}	{{1.21526, 7.69796}, {2.30831, 20.}}	{36.8544, 93.7009}	inf	37.	87.736	{25.3169, 195.868}	151.512	0.369297	newly	32.	36.56	{14., 69.}	50.25	0.221066	Reff	0.99205	0.834027	{0.504435, 1.1124}	24.3548	ar	400.	782.65	{511., 1004.}	95.6725
2	R0	{1.33231, 10.5259}	{1.63036, 12.5215}	{{1.11461, 5.3704}, {2.22731, 18.4377}}	{30.5643, 44.2747}	inf	32.	25.9581	{0.468002, 72.0677}	78.0452	0.688337	newly	17.	10.99	{0., 28.}	68.7647	0.661988	Reff	0.766078	0.679297	{0.383612, 0.921077}	27.1529	ar	467.	589.31	{269., 852.}	46.743
3	R0	{1.2273, 6.76944}	{1.71525, 12.9563}	{{1.13227, 5.72868}, {2.62879, 18.9813}}	{42.8536, 99.2305}	inf	26.	71.3281	{3.10183, 147.459}	210.893	0.549033	newly	25.	35.68	{3., 74.}	87.76	0.422244	Reff	0.996568	0.986593	{0.513548, 1.4299}	26.7786	ar	314.	600.04	{252., 893.}	103.93
4	R0	{1.41215, 17.5742}	{1.55694, 13.1483}	{{1.20115, 7.0488}, {2.20741, 19.6556}}	{19.3891, 29.309}	inf	25.	61.0183	{14.443, 116.923}	163.861	0.389441	newly	9.	27.96	{11., 59.}	217.333	0.443714	Reff	1.19468	0.844638	{0.546396, 1.10925}	29.5527	ar	465.	693.32	{465., 960.}	51.6043
5	R0	{1.42222, 6.15886}	{1.67086, 12.8651}	{{1.14345, 6.78013}, {2.22832, 18.9086}}	{26.5092, 112.833}	inf	35.	57.9355	{4.48368, 133.884}	116.996	0.454212	newly	37.	27.12	{3., 59.}	56.1622	0.440326	Reff	0.924443	0.851305	{0.486748, 1.19116}	27.1686	ar	490.	596.15	{256., 850.}	44.2714
6	R0	{1.24363, 9.95596}	{1.66457, 13.7398}	{{1.11365, 5.98888}, {2.29887, 19.3547}}	{38.533, 53.9136}	inf	46.	73.5799	{4.38824, 173.067}	117.944	0.50191	newly	37.	35.11	{3., 72.}	62.8919	0.447978	Reff	1.06828	1.06545	{0.713138, 1.53048}	23.5949	ar	411.	575.62	{184., 862.}	61.8978
7	R0	{1.36478, 5.96056}	{1.66674, 12.9045}	{{1.13176, 6.18069}, {2.43143, 18.9248}}	{30.1419, 120.459}	inf	43.	63.7634	{3.53587, 138.47}	99.2358	0.433077	newly	48.	30.52	{3., 60.}	51.75	0.45133	Reff	1.03723	0.933239	{0.494644, 1.30898}	27.3331	ar	484.	594.43	{238., 861.}	45.9112
8	R0	{1.36335, 17.6429}	{1.53674, 13.661}	{{1.19672, 6.64462}, {2.02974, 20.}}	{18.8606, 27.6643}	inf	38.	46.1981	{3.33793, 99.9634}	79.6099	0.432466	newly	14.	19.22	{2., 37.}	79.	0.338185	Reff	1.17657	0.761341	{0.491012, 0.962184}	35.2915	ar	484.	694.96	{492., 921.}	44.8802
9	R0	{1.26575, 5.28707}	{1.53734, 13.2715}	{{1.18543, 7.03175}, {1.91479, 18.6019}}	{23.9689, 153.165}	inf	37.	39.4285	{3.99469, 70.8999}	60.4763	0.354357	newly	42.	17.8	{3., 33.}	59.6667	0.540504	Reff	0.882228	0.756522	{0.489945, 0.98336}	19.9776	ar	472.	700.76	{493., 922.}	50.161
10	R0	{1.42942, 9.49905}	{1.62528, 14.2336}	{{1.25207, 7.03512}, {2.10144, 20.}}	{20.9567, 59.7399}	inf	37.	52.0351	{7.48055, 97.9771}	79.8766	0.340408	newly	27.	21.84	{5., 37.}	46.5185	0.331248	Reff	0.86337	0.740612	{0.447807, 1.03187}	22.9252	ar	468.	750.38	{548., 966.}	60.5342
11	R0	{1.46827, 15.0983}	{1.6053, 13.1345}	{{1.20353, 5.45925}, {2.1901, 20.}}	{21.173, 28.762}	inf	35.	34.6376	{0.721084, 84.3351}	72.7698	0.527741	newly	18.	15.23	{1., 36.}	60.6111	0.450902	Reff	1.25831	0.702804	{0.380637, 0.959122}	44.1469	ar	495.	712.44	{484., 960.}	46.1414
12	R0	{1.25751, 13.279}	{1.54594, 12.9058}	{{1.19886, 5.45245}, {2.04606, 19.7498}}	{24.8373, 32.4895}	inf	31.	39.2795	{1.5951, 91.6199}	87.0868	0.471437	newly	18.	16.8	{3., 34.}	58.6667	0.382997	Reff	1.09906	0.748686	{0.430563, 0.975004}	32.0247	ar	366.	690.18	{474., 929.}	88.8306
13	R0	{1.3425, 9.16361}	{1.6759, 12.9132}	{{1.11523, 5.71232}, {2.36153, 18.9813}}	{32.6728, 56.94}	inf	30.	35.8139	{0.977135, 81.7151}	92.0511	0.564031	newly	15.	15.14	{1., 33.}	75.2	0.514356	Reff	0.839063	0.669276	{0.338085, 0.905931}	28.309	ar	442.	610.72	{305., 869.}	52.7873
14	R0	{1.18936, 6.20583}	{1.66976, 12.946}	{{1.11523, 6.31117}, {2.4534, 18.9813}}	{43.3284, 112.903}	inf	36.	52.3165	{5.08892, 109.743}	102.083	0.448802	newly	30.	23.43	{3., 51.}	56.3	0.414594	Reff	0.848014	0.809456	{0.374802, 1.0897}	27.206	ar	382.	590.22	{247., 883.}	69.2565
15	R0	{1.27137, 9.78868}	{1.71488, 14.7877}	{{1.24297, 8.25815}, {2.3898, 20.}}	{36.1249, 60.7794}	inf	42.	75.884	{24.5385, 146.385}	103.129	0.2808	newly	20.	33.95	{13., 56.}	82.85	0.239885	Reff	0.943357	0.974376	{0.629765, 1.2805}	22.7886	ar	496.	782.49	{558., 1025.}	58.2238
16	R0	{1.23102, 13.6452}	{1.64035, 14.4327}	{{1.21756, 7.60472}, {2.28259, 20.}}	{34.0432, 29.6351}	inf	28.	64.7484	{14.8663, 125.632}	149.304	0.381658	newly	7.	27.25	{12., 50.}	295.286	0.545999	Reff	0.984816	0.798064	{0.441593, 1.07428}	24.5792	ar	445.	748.38	{536., 1013.}	68.9034
17	R0	{1.27213, 13.7452}	{1.72709, 14.6657}	{{1.2392, 7.40188}, {2.46179, 20.}}	{36.8781, 28.9654}	inf	25.	80.0829	{27.0806, 139.824}	225.153	0.445748	newly	15.	37.1	{17., 61.}	150.4	0.358136	Reff	1.11566	0.987901	{0.480501, 1.35589}	26.5036	ar	367.	771.3	{542., 994.}	110.163
18	R0	{1.25318, 11.2151}	{1.56593, 13.5966}	{{1.18618, 6.99684}, {2.08783, 19.8127}}	{27.1857, 41.4566}	inf	34.	41.2432	{4.66832, 99.0429}	78.6507	0.390991	newly	18.	18.27	{3., 37.}	58.1667	0.327981	Reff	0.988759	0.721325	{0.442877, 0.940176}	27.9068	ar	416.	704.15	{485., 940.}	69.7284
19	R0	{1.46006, 8.11496}	{1.71862, 13.5576}	{{1.12977, 6.25107}, {2.42687, 19.3547}}	{29.2293, 76.4125}	inf	40.	68.4652	{3.27022, 133.057}	121.199	0.494086	newly	35.	30.08	{2., 64.}	56.	0.437198	Reff	0.995761	0.834898	{0.485138, 1.22408}	27.5731	ar	496.	613.18	{257., 867.}	45.9919
20	R0	{1.14273, 6.71261}	{1.66382, 14.501}	{{1.19399, 7.66849}, {2.51179, 20.}}	{45.7002, 117.392}	inf	37.	66.1851	{19.0287, 123.754}	101.02	0.283564	newly	33.	28.26	{12., 54.}	45.5758	0.25016	Reff	0.861618	0.809151	{0.447106, 1.10255}	24.9802	ar	362.	731.96	{491., 1006.}	102.414
21	R0	{1.42432, 8.23905}	{1.65208, 12.4308}	{{1.11228, 5.32461}, {2.29887, 18.834}}	{28.593, 64.9669}	inf	21.	39.6604	{1.6705, 99.5981}	146.307	0.553195	newly	20.	17.57	{2., 41.}	64.35	0.4628	Reff	1.02409	0.733434	{0.356601, 1.02536}	33.952	ar	405.	580.18	{205., 865.}	63.516
22	R0	{1.29386, 17.863}	{1.65004, 13.838}	{{1.20336, 5.28113}, {2.34884, 20.}}	{30.1193, 28.6183}	inf	30.	75.7697	{11.3205, 158.704}	174.913	0.413398	newly	13.	34.73	{12., 66.}	174.077	0.379586	Reff	1.13601	0.925368	{0.589459, 1.24385}	24.9352	ar	488.	728.47	{479., 983.}	50.8545
23	R0	{1.39274, 13.0167}	{1.62005, 14.5227}	{{1.20471, 8.11803}, {2.15169, 19.9909}}	{23.206, 32.3701}	inf	50.	48.8021	{7.36046, 93.9732}	53.7075	0.334676	newly	23.	20.4	{5., 36.}	44.087	0.279985	Reff	0.997202	0.740892	{0.458009, 1.01479}	27.2691	ar	475.	744.76	{522., 969.}	57.3558
24	R0	{1.32178, 15.8991}	{1.55711, 13.1399}	{{1.19025, 6.98645}, {2.06589, 19.7039}}	{22.5241, 28.3758}	inf	70.	52.7289	{7.41856, 113.828}	55.6445	0.400962	newly	28.	24.13	{7., 47.}	48.6071	0.289713	Reff	1.00059	0.79303	{0.495888, 1.05539}	23.3043	ar	471.	692.86	{467., 951.}	48.862
25	R0	{1.37568, 17.8427}	{1.63446, 14.0931}	{{1.19309, 6.98688}, {2.37607, 19.8952}}	{26.0073, 25.1791}	inf	28.	37.9555	{3.28407, 80.5109}	85.1463	0.399744	newly	9.	16.03	{2., 32.}	111.889	0.390425	Reff	1.11843	0.701699	{0.316415, 0.941245}	37.2771	ar	398.	724.24	{489., 985.}	82.0905
26	R0	{1.20993, 6.4656}	{1.60946, 14.2593}	{{1.18989, 8.02694}, {2.05572, 20.}}	{33.7702, 123.534}	inf	26.	65.3315	{16.1915, 127.57}	168.54	0.392201	newly	24.	27.54	{10., 49.}	51.9167	0.227045	Reff	0.908657	0.830345	{0.536327, 1.10873}	20.4446	ar	345.	729.78	{501., 960.}	111.733
27	R0	{1.20548, 13.8003}	{1.57059, 14.0423}	{{1.19128, 6.49151}, {2.10235, 20.}}	{31.1601, 32.5756}	inf	33.	42.1972	{0.689941, 91.0647}	81.3551	0.478732	newly	20.	17.6	{2., 35.}	55.4	0.42393	Reff	0.947507	0.72069	{0.426023, 0.941015}	25.1672	ar	350.	707.67	{489., 947.}	102.191
28	R0	{1.3176, 14.6594}	{1.63201, 13.2302}	{{1.18527, 5.46077}, {2.35778, 20.}}	{28.5345, 29.3227}	inf	28.	68.0085	{8.71109, 144.981}	164.76	0.418086	newly	16.	31.66	{9., 57.}	115.5	0.322654	Reff	1.18979	0.899679	{0.533099, 1.20631}	29.4191	ar	362.	721.2	{467., 961.}	99.2265
29	R0	{1.28202, 6.23047}	{1.66426, 14.6852}	{{1.20238, 7.40799}, {2.39814, 20.}}	{32.3469, 138.583}	inf	21.	61.4526	{20.245, 120.905}	203.001	0.451052	newly	22.	25.9	{11., 49.}	49.0909	0.224149	Reff	0.937157	0.768811	{0.398433, 1.06642}	24.6643	ar	391.	746.73	{490., 998.}	90.9795
30	R0	{1.28177, 8.83252}	{1.60835, 13.1995}	{{1.11228, 6.77341}, {2.22832, 19.0355}}	{31.9106, 59.6646}	inf	41.	56.4315	{6.78786, 119.374}	92.9954	0.416589	newly	27.	25.97	{5., 54.}	60.8519	0.361093	Reff	0.9562	0.898493	{0.537859, 1.16245}	26.0626	ar	427.	570.92	{236., 853.}	56.0422
31	R0	{1.35886, 5.97708}	{1.71113, 13.4321}	{{1.14292, 6.88118}, {2.29887, 19.0531}}	{32.3494, 127.206}	inf	33.	58.4655	{4.59248, 127.359}	122.258	0.434865	newly	34.	25.81	{4., 58.}	54.9706	0.392864	Reff	0.841134	0.765989	{0.377161, 1.05166}	25.4372	ar	491.	608.06	{259., 866.}	46.9532
32	R0	{1.27601, 17.9804}	{1.52327, 13.5038}	{{1.18318, 6.6576}, {1.92093, 20.}}	{22.216, 28.5174}	inf	45.	55.4198	{2.02521, 117.752}	70.1127	0.389717	newly	24.	24.46	{2., 46.}	54.1667	0.347225	Reff	1.09737	0.828092	{0.53512, 1.06684}	25.2283	ar	445.	697.52	{507., 915.}	56.9213
33	R0	{1.3368, 11.3235}	{1.66977, 14.5586}	{{1.20471, 7.91515}, {2.30063, 20.}}	{28.9895, 41.3744}	inf	59.	71.4884	{17.9485, 132.541}	63.986	0.283219	newly	32.	30.97	{10., 58.}	45.3438	0.238582	Reff	0.974527	0.813216	{0.444688, 1.09888}	23.4273	ar	417.	752.75	{499., 991.}	80.7458
34	R0	{1.21021, 11.3635}	{1.58248, 14.0574}	{{1.19053, 8.18676}, {2.2492, 20.}}	{31.7706, 35.8801}	inf	17.	52.5395	{16.6888, 87.5925}	220.294	0.47686	newly	9.	24.08	{10., 39.}	176.667	0.418094	Reff	1.00689	0.818673	{0.476534, 1.08197}	23.2732	ar	327.	703.2	{443., 976.}	115.046
35	R0	{1.53382, 10.8866}	{1.65127, 14.7955}	{{1.21119, 7.88316}, {2.3559, 20.}}	{22.6961, 46.8567}	inf	51.	78.1405	{23.4034, 142.364}	82.3866	0.279242	newly	30.	33.67	{14., 62.}	49.9667	0.214557	Reff	1.1565	0.857836	{0.52242, 1.12022}	27.6624	ar	476.	742.17	{499., 1009.}	57.0903
36	R0	{1.20423, 6.83844}	{1.68293, 13.1351}	{{1.14345, 6.18069}, {2.24242, 19.0531}}	{41.8263, 99.4593}	inf	51.	68.6707	{2.53854, 154.409}	92.5248	0.458281	newly	46.	30.23	{3., 64.}	53.3696	0.444108	Reff	0.814059	0.867273	{0.537859, 1.18367}	25.8918	ar	416.	593.77	{249., 861.}	60.7716
37	R0	{1.43629, 10.1548}	{1.5811, 14.0344}	{{1.21549, 7.82721}, {2.01042, 20.}}	{19.0136, 49.1597}	inf	42.	66.9458	{15.0787, 125.771}	90.5313	0.317808	newly	33.	29.93	{11., 56.}	44.0909	0.253438	Reff	1.10594	0.873524	{0.57012, 1.15665}	23.3898	ar	367.	720.95	{502., 929.}	96.9401
38	R0	{1.37504, 8.93428}	{1.67501, 12.7475}	{{1.14292, 5.15387}, {2.25647, 19.0269}}	{29.9852, 60.542}	inf	75.	63.2471	{2.47175, 132.03}	69.4206	0.58676	newly	60.	27.9	{1., 63.}	57.2667	0.636798	Reff	0.897901	0.851297	{0.52444, 1.17085}	25.48	ar	483.	580.75	{188., 861.}	46.4161
39	R0	{1.51637, 9.36742}	{1.63296, 12.9512}	{{1.11461, 6.18069}, {2.27582, 18.9248}}	{25.603, 52.3432}	inf	30.	50.0275	{4.29022, 110.331}	120.286	0.458355	newly	16.	22.26	{3., 49.}	87.75	0.377787	Reff	1.019	0.800105	{0.433202, 1.08248}	30.526	ar	441.	581.11	{259., 868.}	51.6757
40	R0	{1.23658, 9.82869}	{1.67, 12.7586}	{{1.13227, 5.32185}, {2.22832, 18.9813}}	{38.4408, 50.4619}	inf	71.	65.3728	{2.45583, 148.334}	73.4098	0.545681	newly	51.	30.21	{2., 65.}	54.6863	0.540903	Reff	0.868079	0.915205	{0.583978, 1.27995}	27.2462	ar	414.	586.05	{221., 866.}	63.0072
41	R0	{1.24247, 5.4242}	{1.70518, 13.3285}	{{1.12977, 5.15387}, {2.24606, 18.9813}}	{40.797, 152.228}	inf	30.	72.4268	{2.57648, 155.317}	181.43	0.550227	newly	38.	33.59	{3., 69.}	57.2895	0.45108	Reff	0.956702	0.959808	{0.575745, 1.51022}	27.8566	ar	345.	630.44	{316., 861.}	90.1913
42	R0	{1.28208, 4.40223}	{1.7477, 13.0239}	{{1.14345, 6.05643}, {2.46246, 19.0531}}	{40.1976, 197.504}	inf	25.	79.3386	{2.45583, 174.474}	250.547	0.57478	newly	44.	36.33	{3., 73.}	52.7955	0.411529	Reff	0.951303	0.897365	{0.555024, 1.32359}	25.1316	ar	339.	615.85	{225., 890.}	96.2507
43	R0	{1.56773, 6.50474}	{1.87686, 12.8188}	{{1.21457, 6.62607}, {2.59755, 17.6755}}	{30.5249, 102.093}	inf	43.	94.2496	{13.0329, 203.475}	151.246	0.455286	newly	49.	45.62	{8., 81.}	44.8163	0.317603	Reff	0.981399	0.915504	{0.483976, 1.4114}	28.0579	ar	498.	674.65	{260., 902.}	51.9659
44	R0	{1.10489, 6.01941}	{1.57045, 14.0636}	{{1.17662, 7.37338}, {2.19074, 19.9982}}	{42.1364, 137.06}	inf	33.	50.8529	{15.2087, 93.4133}	80.382	0.309246	newly	35.	22.42	{10., 38.}	43.9429	0.333561	Reff	0.79994	0.761155	{0.420202, 0.980384}	20.9554	ar	346.	702.35	{504., 977.}	102.991
45	R0	{1.25888, 14.1926}	{1.54814, 14.9898}	{{1.18202, 9.29344}, {2.01204, 20.}}	{24.9963, 24.1544}	inf	65.	53.0908	{18.4173, 99.8874}	49.7014	0.299669	newly	24.	22.54	{8., 41.}	46.5833	0.236382	Reff	0.815754	0.769599	{0.508852, 1.00886}	19.0966	ar	471.	726.08	{502., 944.}	55.1975
46	R0	{1.45292, 16.616}	{1.69539, 14.832}	{{1.22866, 8.33176}, {2.43288, 19.8504}}	{25.531, 21.7437}	inf	45.	86.2712	{30.9551, 159.323}	104.022	0.274668	newly	23.	39.09	{17., 65.}	81.1739	0.242512	Reff	1.20738	0.960659	{0.526115, 1.17423}	25.6572	ar	494.	753.79	{491., 1007.}	54.4717
47	R0	{1.22528, 16.6033}	{1.5439, 13.8807}	{{1.19204, 7.12632}, {2.16344, 19.9232}}	{27.337, 25.5107}	inf	28.	61.7969	{13.8208, 115.35}	140.449	0.373828	newly	12.	27.68	{8., 51.}	143.667	0.359558	Reff	1.1101	0.868661	{0.575803, 1.10618}	24.319	ar	368.	695.16	{465., 933.}	88.9022
48	R0	{1.1669, 6.92715}	{1.56658, 14.2328}	{{1.19493, 7.9966}, {2.10946, 20.}}	{34.4745, 107.056}	inf	37.	41.898	{10.2203, 82.3362}	59.8414	0.312766	newly	38.	18.04	{4., 31.}	54.3684	0.442115	Reff	0.839001	0.76323	{0.404183, 1.0078}	20.5857	ar	416.	709.67	{509., 947.}	70.762
49	R0	{1.32749, 16.0887}	{1.51398, 13.5479}	{{1.18797, 6.98109}, {1.9746, 18.9573}}	{20.4856, 25.5987}	inf	27.	58.0189	{12.208, 120.751}	136.68	0.377994	newly	11.	26.19	{8., 48.}	149.909	0.373541	Reff	1.17085	0.854096	{0.589173, 1.06495}	27.7248	ar	353.	675.21	{456., 896.}	91.2776
50	R0	{1.29636, 15.2609}	{1.62212, 14.4516}	{{1.20872, 7.40322}, {2.36128, 19.9456}}	{28.2685, 25.8093}	inf	42.	52.5708	{8.10327, 104.505}	67.7618	0.335984	newly	19.	21.88	{5., 36.}	53.2632	0.279003	Reff	0.917823	0.736554	{0.401656, 0.980801}	23.3562	ar	470.	734.48	{493., 987.}	57.9872
